# Antibiotic consumption in post-acute care and geriatric hospitals in Israel, 2014

**DOI:** 10.1186/2047-2994-4-S1-P185

**Published:** 2015-06-16

**Authors:** E Temkin, M Schwaber, S Masawra, Y Carmeli

**Affiliations:** 1 National Center for Infection Control, Ministry of Health, Israel

## Introduction

In 2012, the Israeli Ministry of Health issued national guidelines for antibiotic stewardship. The guidelines require that all general hospitals, post-acute care hospitals and the ambulatory care sectors of health maintenance organizations report annual consumption of antibiotics. Data regarding antibiotic use in post-acute care hospitals are scarce.

## Objectives

To describe antibiotic consumption in post-acute care and geriatric hospitals in Israel in 2014.

## Methods

Hospitals used their computerized pharmacy databases to report all systemic antibacterials (WHO Anatomical Therapeutic Chemical class J01) dispensed. Hospitals also reported patient days, by ward, for 2014. We calculated antibiotic consumption as Defined Daily Doses (DDDs) per 1000 patient-days (PD). To overcome differences in case mix between institutions, we limited our analysis to 4 common ward types: ventilation /respiratory rehabilitation, sub-acute care, rehabilitation, and complex nursing.

## Results

Seven of Israel’s 15 post-acute care and geriatric hospitals reported at the ward level. Median use of systemic antibacterials in DDD/1000 PDs was 206 (range: 130-313) in complex nursing wards, 232 (range: 172-323) in ventilation wards, 244 (range: 154-379) in rehabilitation wards, and 348 (range: 272-600) in sub-acute wards. (By comparison, median use in medical wards in general hospitals in 2014 was 933, range: 629-1340.) The most commonly used antibiotic group was penicillins with beta-lactamase inhibitors in ventilation and complex nursing wards and cephalosporins in sub-acute and rehabilitation wards. Fluoroquinolones accounted for 14% -19% of all antibiotic use, depending on ward type. Individual hospitals were consistently either below the median or above the median for all ward types.

## Conclusion

Antibiotic consumption in the major ward types of post-acute care and geriatric hospitals in Israel is substantial, amounting to over one-fourth of the amount used in acute care hospitals’ medical wards. Our data underestimate total antibiotic use in this patient population because patients may be temporarily transferred to general hospitals for treatment of complex infections. The consistently high or low use in each hospital suggests that antibiotic use is influenced by institutional culture or formal policies that dictate a permissive or restrictive approach.

## Disclosure of interest

None declared.

